# A rare case of renal thrombotic microangiopathy associated with Castleman’s disease

**DOI:** 10.1186/s12882-017-0472-2

**Published:** 2017-02-10

**Authors:** Anubha Mutneja, L. Nicholas Cossey, Helen Liapis, Ying Maggie Chen

**Affiliations:** 1 Division of Nephrology 8126, Department of Internal Medicine, Washington University School of Medicine, 660 S. Euclid Ave., St. Louis, MO 63110 USA; 2 Arkana Laboratories, Little Rock, AR USA; 3 Department of Pathology & Immunology, Washington University School of Medicine, St. Louis, MO USA

**Keywords:** Thrombotic microangiopathy, Castleman’s disease, VEGF, Podocytes

## Abstract

**Background:**

Castleman’s disease (CD) is an uncommon, heterogeneous lympho-proliferative disorder leading to high circulating levels of interleukin-6 (IL-6) and vascular endothelial growth factor (VEGF). Renal involvement has been only described in a limited number of small studies. Herein, we report a rare case of renal thrombotic microangiopathy (TMA) associated with CD and investigate the podocyte expression of VEGF in the renal biopsy prior to initiation of treatment.

**Case presentation:**

An 18-year-old male presented with fever, diarrhea, diffuse lymphadenopathy, ascites and acute kidney injury. Laboratory tests for hemolytic uremic syndrome and thrombotic thrombocytopenic purpura were negative. The kidney biopsy showed TMA. An excisional lymph node biopsy was consistent with CD, plasma cell variant. Immunofluorescence staining showed suppressed podocyte VEGF expression. Chemotherapy that inhibits production of inflammatory mediators including IL-6 and VEGF led to complete recovery of renal function.

**Conclusions:**

Our case illustrates a rare renal histological feature of CD. IL-6 and VEGF are postulated to suppress glomerular VEGF expression, thereby causing renal TMA. Therapy directed against these inflammatory mediators may have important therapeutic implications.

## Background

Castleman’s disease (CD), first described in 1956, is an uncommon lymphoproliferative disorder characterized by focal or generalized lymphadenopathy [1]. Clinically, this group of lymphoproliferative disorders is classified as unicentric CD (localized lymph node involvement, UCD), or multicentric CD (diffuse lymph node involvement, MCD). Systemic manifestations commonly associated with MCD include fever, weight loss, hepatosplenomegaly, ascites, edema, and anemia. Histologically, there are three different variants of CD: hyaline-vascular variant, plasma cell variant and mixed variant. The hyaline-vascular variant accounts for 90% of CD and is characterized by small hyaline vascular follicles and interfollicular capillary proliferation. It is typically associated with UCD and most patients exhibit no symptoms. The plasma cell variant (9%) is characterized by hyperplastic follicles with interfollicular sheets of plasma cells and is often associated with MCD.

Thrombotic microangiopathy (TMA) is a histopathological term for microangiopathic hemolytic anemia, thrombocytopenia, and renal microvascular thrombosis. Diagnostic TMA features include endothelial swelling in glomerular capillaries and arterioles, mesangiolysis (dissolution or attenuation of mesangial matrix and degeneration of mesangial cells), and glomerular basement membrane double contours (a feature of glomerular basement membrane remodeling). The latter can be seen without thrombi, which suggests subacute or chronic TMA. Hemolytic uremic syndrome (HUS) and thrombotic thrombocytopenic purpura (TTP) are two major clinical entities comprising TMA with predominantly renal manifestations in the former whereas neurological and systemic manifestations in the latter, although overlap exists and differentiation of HUS from TTP is not always possible.

Here, we present a complicated case of renal TMA in a MCD patient in the absence of TTP/HUS. In the presented patient, decreased podocyte expression of vascular endothelial growth factor (VEGF) is linked to TMA associated with CD.

## Case presentation

A 19-year-old Caucasian male was transferred to Washington University Barnes-Jewish Hospital for evaluation of acute renal failure (ARF) and previously diagnosed TMA on renal biopsy. He presented with anasarca, mild proteinuria, and diffuse lymphadenopathy. He was initially admitted to a community hospital with diarrhea, nausea, vomiting, generalized swelling, and low grade fever for 3 weeks. There was no history of recent travel, medication use or change in his home environment. While in the community hospital, he was noted to have new onset of thrombocytopenia and anemia without evidence of hemolysis or schistocytes. Stool *Shiga* toxin was negative, and stool cultures were negative for *E. coli* O157:H7, *Salmonella*, *Shigella*, and *Campylobacter*. Ascites was negative for peritonitis and malignancy, and an inguinal lymph node core biopsy was reported as nonspecific reactive hyperplasia.

**Fig. 1:**
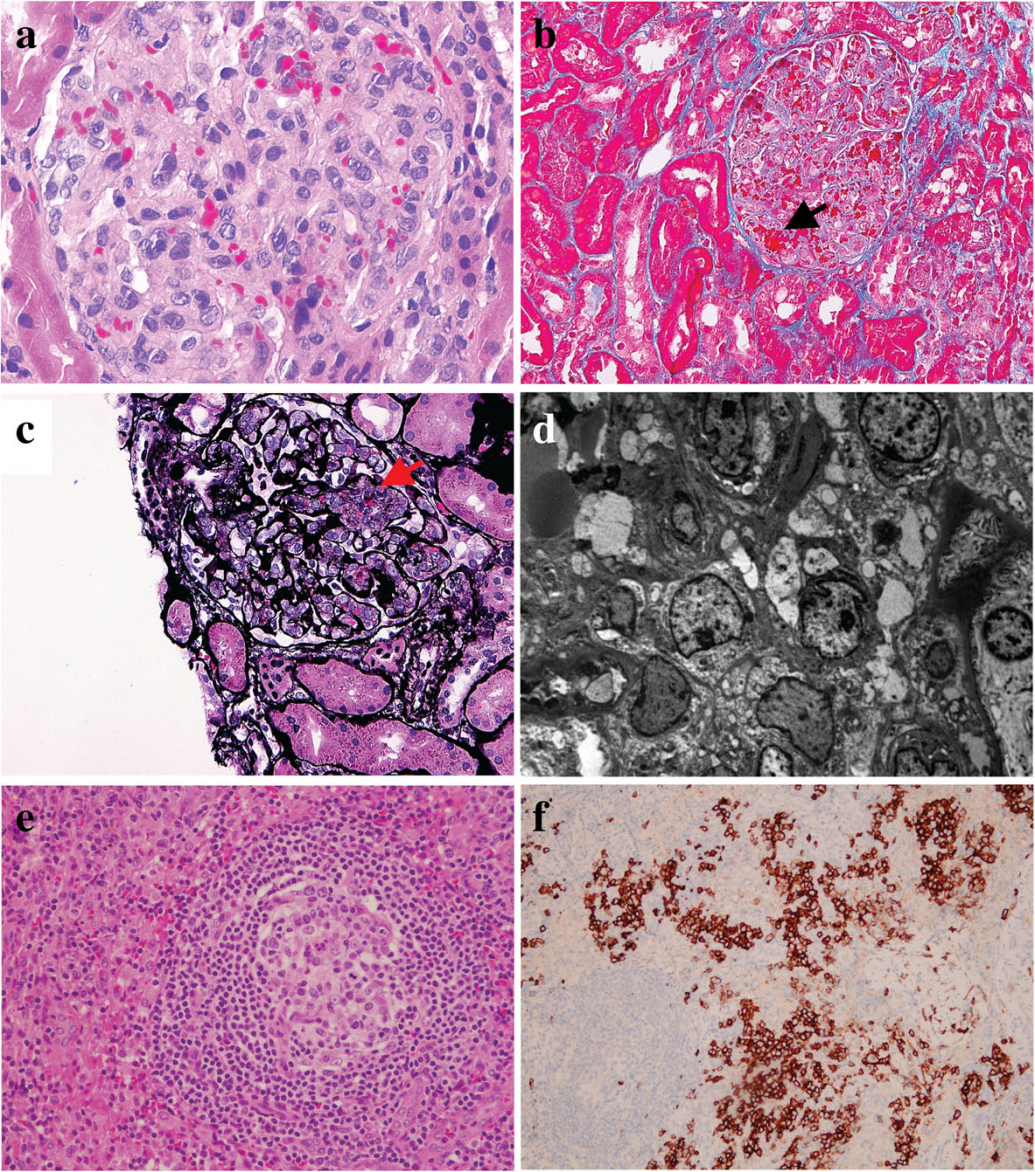
Histopathological findings in renal and the inguinal lymph node biopsies from the patient with MCD. Renal (**a**-**d**) and lymph node biopsies (**e**-**f**) at the onset of disease. **a** Diffuse endocapillary proliferation and endothelial swelling (Hematoxylin and eosin stain [H&E], ×400). **b** Thrombi within capillary loops (*arrow*; Masson trichrome, ×200). **c** Mesangiolysis (*arrow*) and double contour (Jones Methenamine Silver, ×200). **d** Transmission electron microscopy showed diffuse endothelial swelling with obliteration of capillary lumina and mesangial interposition (×4000). **e** Hyalinization of the germinal center and concentric layering of peripheral lymphocytes (H&E, ×200). **f** Plasmacytosis highlighted with CD138 staining in the interfollicular space is characteristic of plasma cell CD type (×200)

Renal biopsy was performed and showed diffuse endothelial swelling, mesangiolysis and thrombi within capillary loops (Fig. 1, a-d). Occasional double contours were present (Fig. 1c). There were no crescents, fibrinoid necrosis, or significant fibrosis. Immunofluorescence (IF) was negative for immune complex deposits. TMA was diagnosed. ADAMTS 13 (a disintegrin and metalloproteinase with a thrombospondin type 1 motif, member 13) activity was decreased (20%; normal levels, ≥67%). 10 sessions of plasmapheresis performed in the community hospital did not improve his anemia, thrombocytopenia and kidney dysfunction.

On admission, tachycardia (109/min), hypertension (148/86 mmHg), and normal body temperature were observed. Conjunctivas were pale but not icteric. Respiratory sounds were decreased bilaterally. The abdomen was distended with free fluid. Lower extremities showed 1+ pedal edema, and lymph nodes were palpable in the axillary and inguinal regions.

Laboratory evaluation revealed microcytic anemia (hemoglobin 8.1 g/dL) and thrombocytopenia (platelets, 75,000/mm3) with lactate dehydrogenase and haptoglobin being within normal range. The repeated peripheral smear did not show schistocytes. Renal function had deteriorated (blood urea nitrogen [BUN], 27 mg/dL; serum creatinine [Cr], 2.18 mg/dL). The patient had normal liver function but with decreased total protein (6.2 g/dL) and albumin (3.0 g/dL). Complement levels were normal. Acute phase reactants including erythrocyte sedimentation rate (ESR, 59 mm/h; normal levels, <12 mm/h), C-reactive protein (CRP, 99.4 mg/L; normal levels, <9.9 mg/L) and ferritin (348 ng/ml; normal levels, <322 ng/ml) were elevated. An increased IgG of 1560 mg/dl (normal range: 700–1450 mg/dl) with normal levels of IgA and IgM was found. There was no monoclonal peak on immunoelectrophoresis in either serum or urine. Autoantibodies including antinuclear antibody (titer, 1:640) and anti-SS-A and anti-SS-B were positive. Additionally, serum lupus anticoagulant was positive, but β2 glycoprotein and anti-cardiolipin antibody were negative. Test results for hepatitis virus A, B and C, human immunodeficiency virus, Epstein-Barr virus, and cytomegalovirus were negative. Urinalysis showed microhematuria without any casts and non-nephrotic range proteinuria (683 mg/24 h).

Renal ultrasound showed normal size and echogenicity of both kidneys. Computed tomography (CT) of the chest, abdomen and pelvis showed retroperitoneal, mesenteric, pelvic, axillary and mediastinal lymphadenopathy as well as hepatosplenomegaly, moderate ascites and small bilateral pleural effusions.

**Fig. 2:**
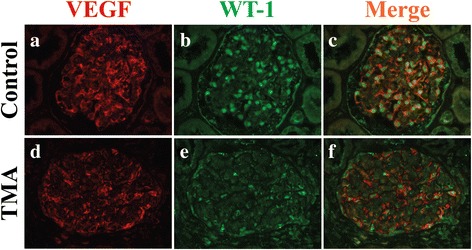
Podocyte VEGF expression in healthy control kidney and renal biopsy from the CD-associated TMA patient. Formalin-fixed paraffin kidney sections from control (**a-c**) and patient (**d-f**) were examined by dual IF staining of VEGF (*red*, **a** and **d**) and podocyte nucleus marker WT-1 (*green*, **b** and **e**). Merged images for VEGF and WT-1 in **c** and **f**. Note the decreased podocyte VEGF expression in CD-associated renal TMA. Original magnifications: ×400

An excisional lymph node biopsy performed in our hospital showed follicular hyperplasia and diffuse interfollicular plasma cell infiltrate (Fig. 1 e–f). Some of the lymphoid follicles showed hyalinization of germinal centers and concentric layering of peripheral lymphocytes (onion-skinning) in the mantle area (Fig. 1e). Immunohistochemistry revealed interfollicular plasmacytosis demonstrated by CD138 staining (Fig. 1f). HHV-8 immunostaining was negative. The patient’s diagnosis was consistent with MCD, plasma cell variant. High circulating level of VEGF (343 pg/ml; normal levels, <86 pg/ml) was detected in this patient, a characteristic feature of CD. Notably, double IF staining for VEGF and the podocyte nucleus marker WT-1 showed that podocyte VEGF expression was reduced in this patient compared to healthy control (Fig. 2).

The patient was started on chemotherapy with Rituximab, Etoposide, Cyclophosphamide, Vincristine and Prednisone for 6 cycles. Within a week of initiation of chemotherapy, his renal function normalized. His clinical symptoms such as fever, ascites and lymphadenopathy started improving. Anemia and thrombocytopenia resolved, and markers of inflammation such as ESR and CRP normalized within the first few weeks. The patient has been free of symptoms for 2 years without taking any medication.

## Discussion

CD is a rare lymphoproliferative disorder. Renal involvement has been only described in a limited number of small studies. Based on a study conducted in a large CD cohort of Chinese patients, renal involvement in CD is about 25% [2], particularly seen in patients with MCD plasma cell or mixed variant [2, 3]. ARF represents the most common renal presentation, and glomerular pathologies majorly include AA amyloidosis [3, 4], TMA [2, 3, 5] and membranoproliferative glomerulonephritis [6]. Other renal pathological findings include mesangioproliferative glomerulonephritis, interstitial nephritis [7], membranous nephropathy [8], crescentic glomerulonephritis [2, 9, 10], minimal change disease [2] and focal segmental glomerulosclerosis [3]. Consistent with previous studies, our patient developed renal TMA in MCD plasma cell variant, the aggressive type of CD.

The dysregulated production of VEGF and interleukin-6 (IL-6) is implicated to play a central role in the pathogenesis of CD. Proliferating plasma cells in lymph nodes cause overproduction of both cytokines leading to elevated systemic levels [11, 12]. VEGF exerts potent pro-inflammatory effect and increases blood vessel permeability, which explains pleural effusion, ascites, and generalized edema in our patient. IL-6 has pleotropic effects, such as promoting B-cell differentiation to immunoglobulin-producing plasma cells, stimulating hepatocyte to synthesize acute-phase proteins, and inducing dominance of Th17 cells over Tregs to disrupt immune tolerance [13]. It has been shown that mice overexpressing IL-6 produce a syndrome resembling CD [14]. In our patient, IL-6 accounts for a variety of clinical symptoms including generalized lymphadenopathy and hepatosplenomegaly, polyclonal hypergammaglobulinemia, increased levels of ESR, CRP and ferritin, anemia, and overproduction of autoantibodies.

Diffuse endothelial swelling, mesangiolysis and capillary loop thrombi in combination with podocyte VEGF downregulation in our patient resemble renal TMA seen in cancer patients treated with VEGF inhibitors [15] or preeclampsia patients with increased circulating level of soluble fms-like tyrosine kinase 1 (sFlt1), a soluble VEGF receptor and an antagonist of VEGF [16]. VEGF is a vascular growth factor for vasculogenesis and angiogenesis during development and in disease through regulation of vascular permeability, endothelial cell migration, proliferation, and cell survival [17]. VEGF released by podocytes binds to VEGF receptors on glomerular endothelial cells, resulting in receptor tyrosine phosphorylation and signal transduction. It has been demonstrated that tight regulation of glomerular VEGF signaling is critical for establishment and maintenance of the glomerular filtration barrier. Moreover, it has been shown that podocyte VEGF deletion in adult mice is sufficient to trigger TMA by utilizing podocyte-specific VEGF knockout mice [15, 18]. Previously, Karoui et al. reported decreased glomerular VEGF expression in a MCD patient with renal TMA [3]. However, co-localization staining of glomerular VEGF with any glomerular cell-specific marker was not performed. The serum level of VEGF from the same affected patient was also not examined. For the first time, we performed dual IF staining of VEGF with the podocyte nucleus marker WT-1 in the kidney biopsy and correlated with serum VEGF level in our patient. Our study showed podocyte VEGF downregulation associated with markedly elevated serum VEGF before chemotherapy. The question of how persistent VEGF and IL-6 overproduction leads to deficient podocyte VEGF expression remains unresolved.

It is of note that not every CD patient develops renal TMA. Genetic or acquired dysregulation of ADAMTS13 or the complement alternative pathway can predispose patients to develop TMA. Deficiency of ADAMTS13, owing to mutations in the *ADAMTS13* gene or autoantibodies that inhibit ADAMTS13 activity, can cause idiopathic TTP. It is not unusual for ADAMTS13 to be decreased in TMA without TTP. In TTP, ADAMTS13 activity is typically <5% [19]. Therefore the TMA in our patient does not appear to be caused by TTP, particularly in the absence of hemolysis, schistocytes and neurological symptoms. Dysfunction of the complement alternative pathway, caused by mutations in complement factor H, I, B, or membrane cofactor protein or by autoantibodies against factor H, can result in complement-mediated TMA [20, 21]. The presented patient may have mild complement abnormalities that increase his susceptibility to develop renal TMA associated with CD.

In summary, we discuss a rare case of MCD patient presenting with ARF, mild proteinuria and renal TMA. The renal injury is associated with inhibited expression of podocyte VEGF. In this patient, an excellent renal response to chemotherapy was achieved. Reagents that suppress overproduction of IL-6 and VEGF may lead to highly-targeted treatments in CD. In addition, further investigation to delineate the mechanism(s) involved in podocyte VEGF downregulation will lead to discovery of specific target molecules for the treatment of renal TMA seen in CD.

## Abbreviations

ADAMTS 13: A disintegrin and metalloproteinase with a thrombospondin type 1 motif, member 13
ARF: Acute renal failure
BUN: Blood urea nitrogen
CD: Castleman’s disease
Cr: Creatinine
CRP: C-reactive protein
CT: Computed tomography
ESR: Erythrocyte sedimentation rate
H&E: Hematoxylin and eosin stain
HUS: Hemolytic uremic syndrome
IF: Immunofluorescence
IL-6: Interleukin-6
MCD: Multicentric CD
sFlt1: Soluble fms-like tyrosine kinase 1
TMA: Thrombotic microangiopathy
TTP: Thrombotic thrombocytopenic purpura
UCD: Unicentric CD
VEGF: Vascular endothelial growth factor

## References

[1] Castleman B, Iverson L, Menendez VP (1956). Localized mediastinal lymphnode hyperplasia resembling thymoma. Cancer.

[2] Xu D, Lv J, Dong Y, Wang S, Su T, Zhou F, Zou W, Zhao M, Zhang H (2012). Renal involvement in a large cohort of Chinese patients with Castleman disease. Nephrol Dial Transplant.

[3] El Karoui K, Vuiblet V, Dion D, Izzedine H, Guitard J, Frimat L, Delahousse M, Remy P, Boffa JJ, Pillebout E (2011). Renal involvement in Castleman disease. Nephrol Dial Transplant.

[4] Funabiki K, Kaneko S, Terajima M, Tomita H, Kawano Y, Tomino Y (1998). A case of multicentric Castleman’s disease associated with renal amyloidosis and pure red cell aplasia. Am J Nephrol.

[5] Seida A, Wada J, Morita Y, Baba M, Eguchi J, Nishimoto N, Okino T, Ichimura K, Yoshino T, Makino H (2004). Multicentric Castleman’s disease associated with glomerular microangiopathy and MPGN-like lesion: does vascular endothelial cell-derived growth factor play causative or protective roles in renal injury?. Am J Kidney Dis.

[6] Said R, Tarawneh M (1992). Membranoproliferative glomerulonephritis associated with multicentric angiofollicular lymph node hyperplasia. Case report and review of the literature. Am J Nephrol.

[7] Summerfield GP, Taylor W, Bellingham AJ, Goldsmith HJ (1983). Hyaline-vascular variant of angiofollicular lymph node hyperplasia with systemic manifestations and response to corticosteroids. J Clin Pathol.

[8] Weisenburger DD (1979). Membranous nephropathy. Its association with multicentric angiofollicular lymph node hyperplasia. Arch Pathol Lab Med.

[9] Furuichi K, Wada T, Shimizu M, Segawa C, Ohta S, Takasawa K, Kobayashi K, Yokoyama H (1998). Antimyeloperoxidase-antibody-positive rapidly progressive glomerulonephritis associated with Castleman’s disease. Nephrol Dial Transplant.

[10] Tsukamoto Y, Hanada N, Nomura Y, Hiki Y, Kasai K, Shigematsu H, Kobayashi Y (1991). Rapidly progressive renal failure associated with angiofollicular lymph node hyperplasia. Am J Nephrol.

[11] Nishi J, Arimura K, Utsunomiya A, Yonezawa S, Kawakami K, Maeno N, Ijichi O, Ikarimoto N, Nakata M, Kitajima I (1999). Expression of vascular endothelial growth factor in sera and lymph nodes of the plasma cell type of Castleman’s disease. Br J Haematol.

[12] Yoshizaki K, Matsuda T, Nishimoto N, Kuritani T, Taeho L, Aozasa K, Nakahata T, Kawai H, Tagoh H, Komori T (1989). Pathogenic significance of interleukin-6 (IL-6/BSF-2) in Castleman’s disease. Blood.

[13] Tanaka T, Kishimoto T (2014). The biology and medical implications of interleukin-6. Cancer Immunol Res.

[14] Brandt SJ, Bodine DM, Dunbar CE, Nienhuis AW (1990). Dysregulated interleukin 6 expression produces a syndrome resembling Castleman’s disease in mice. J Clin Invest.

[15] Eremina V, Jefferson JA, Kowalewska J, Hochster H, Haas M, Weisstuch J, Richardson C, Kopp JB, Kabir MG, Backx PH (2008). VEGF inhibition and renal thrombotic microangiopathy. N Engl J Med.

[16] Maynard SE, Min JY, Merchan J, Lim KH, Li J, Mondal S, Libermann TA, Morgan JP, Sellke FW, Stillman IE (2003). Excess placental soluble fms-like tyrosine kinase 1 (sFlt1) may contribute to endothelial dysfunction, hypertension, and proteinuria in preeclampsia. J Clin Invest.

[17] Eremina V, Quaggin SE (2010). Biology of anti-angiogenic therapy-induced thrombotic microangiopathy. Semin Nephrol.

[18] Eremina V, Sood M, Haigh J, Nagy A, Lajoie G, Ferrara N, Gerber HP, Kikkawa Y, Miner JH, Quaggin SE (2003). Glomerular-specific alterations of VEGF-A expression lead to distinct congenital and acquired renal diseases. J Clin Invest.

[19] Go RS, Winters JL, Leung N, Murray DL, Willrich MA, Abraham RS, Amer H, Hogan WJ, Marshall AL, Sethi S et al. Thrombotic Microangiopathy Care Pathway: A Consensus Statement for the Mayo Clinic Complement Alternative Pathway-Thrombotic Microangiopathy (CAP-TMA) Disease-Oriented Group. Mayo Clin Proc. 2016;91(9):1189–211. 10.1016/j.mayocp.2016.05.015 27497856

[20] De Vriese AS, Sethi S, Van Praet J, Nath KA, Fervenza FC (2015). Kidney Disease Caused by Dysregulation of the Complement Alternative Pathway: An Etiologic Approach. J Am Soc Nephrol.

[21] Zheng XL, Sadler JE (2008). Pathogenesis of thrombotic microangiopathies. Annu Rev Pathol.

